# A Case of Postaxial Polydactyly Managed Under Local Anesthesia

**DOI:** 10.7759/cureus.64626

**Published:** 2024-07-15

**Authors:** Joben Samuel, Pankaj Gharde, Prakher Shrivastava, Dheeraj Surya

**Affiliations:** 1 General Surgery, Jawaharlal Nehru Medical College, Datta Meghe Institute of Higher Education and Research, Wardha, IND

**Keywords:** non-syndromic polydactyly, syndromic polydactyly, extra digit of the hands, local anesthesia, post axial polydactyly, supernumerary digits

## Abstract

Polydactyly is a common occurrence, observed as the presence of extra digit/s in the hands and feet. It can be categorized into preaxial, postaxial, and mesoaxial forms based on the location of the additional digit. In most instances only a single extra digit is present, research reports with more than one extra digit have been published. Most common management includes surgical excision under the influence of general anesthesia. An alternative approach by removing the pre-axial and post-axial supernumerary digit is carried out under local anesthesia in infants and small children, providing the additional benefit of fewer post-procedural complications. This is a case of a 5-month-old male child, with post-axial polydactyly of the left hand. He was managed by excision of the extra digit under the influence of local anesthesia. The patient recovered well and was discharged 3 days after the procedure with the advice of monthly follow-up until 3 months.

## Introduction

An autosomal inherited trait with differential expression level is noted as syndromic and non-syndromic polydactyly. Polydactyly appears in numerous forms, including extra digits on the hands and feet [1]. It is also termed hyperdactyly or supernumerary digit [2,3]. It has been categorized into two subtypes: Type A and Type B. Type A involves well-formed digits with osseous connections with the remaining of the hand and Type B is observed when the additional digits are connected to the body without the osseous connection and the digits are mostly non-functional [2]. There are six genes (GLI1, GLI3, IQCE, MIPOL1, PITX1, and ZNF141) and 10 loci found to be associated with non-syndromic polydactyly [2-4]. Polydactyly, particularly postaxial type, often involves supernumerary digits located adjacent to the fifth digit of the hand or foot. If left untreated into early childhood, common complications may include functional impairment due to altered biomechanics, difficulty with footwear, and potential psychological impacts as the child grows older. Addressing these points will provide a more comprehensive understanding of the implications and management strategies associated with postaxial polydactyly. This is the case of a 5-month-old male child presenting with an extra digit in his left hand since birth.

## Case presentation

A mother presented with her 5-month-old male child with the chief complaint of an extra digit on the left hand.

**Figure 1:**
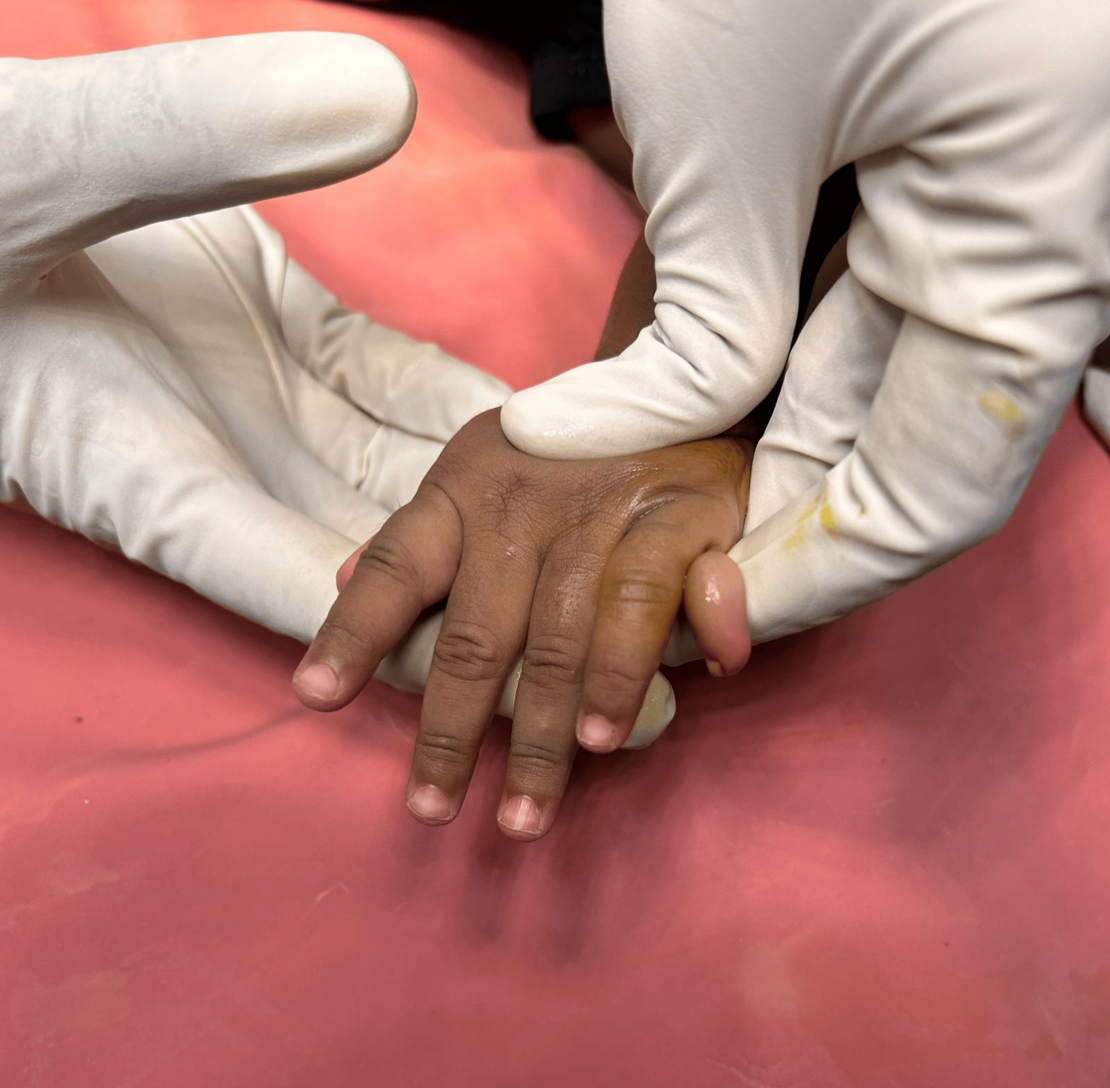
Physical presentation of the left hand

Physical examination revealed postaxial polydactyly of the left hand, characterized by an extra digit attached by a narrow pedicle. These abnormalities were noticed at birth with no other abnormalities. The child took birth under full-term monitored pregnancy. The parents have two children, with this one being the younger one. The elder child did not have any physical abnormality or congenital disorder. The child was delivered by spontaneous vaginal delivery. No other illness was reported in the mother during pregnancy. There was no use of any other drugs apart from routine antenatal drugs during pregnancy. There was no history of any radiation exposure. After a thorough physical examination, the extra finger was found to be postaxial hanging with a stalk without any bony abnormality on clinical examination (Figure 1).

**Figure 2:**
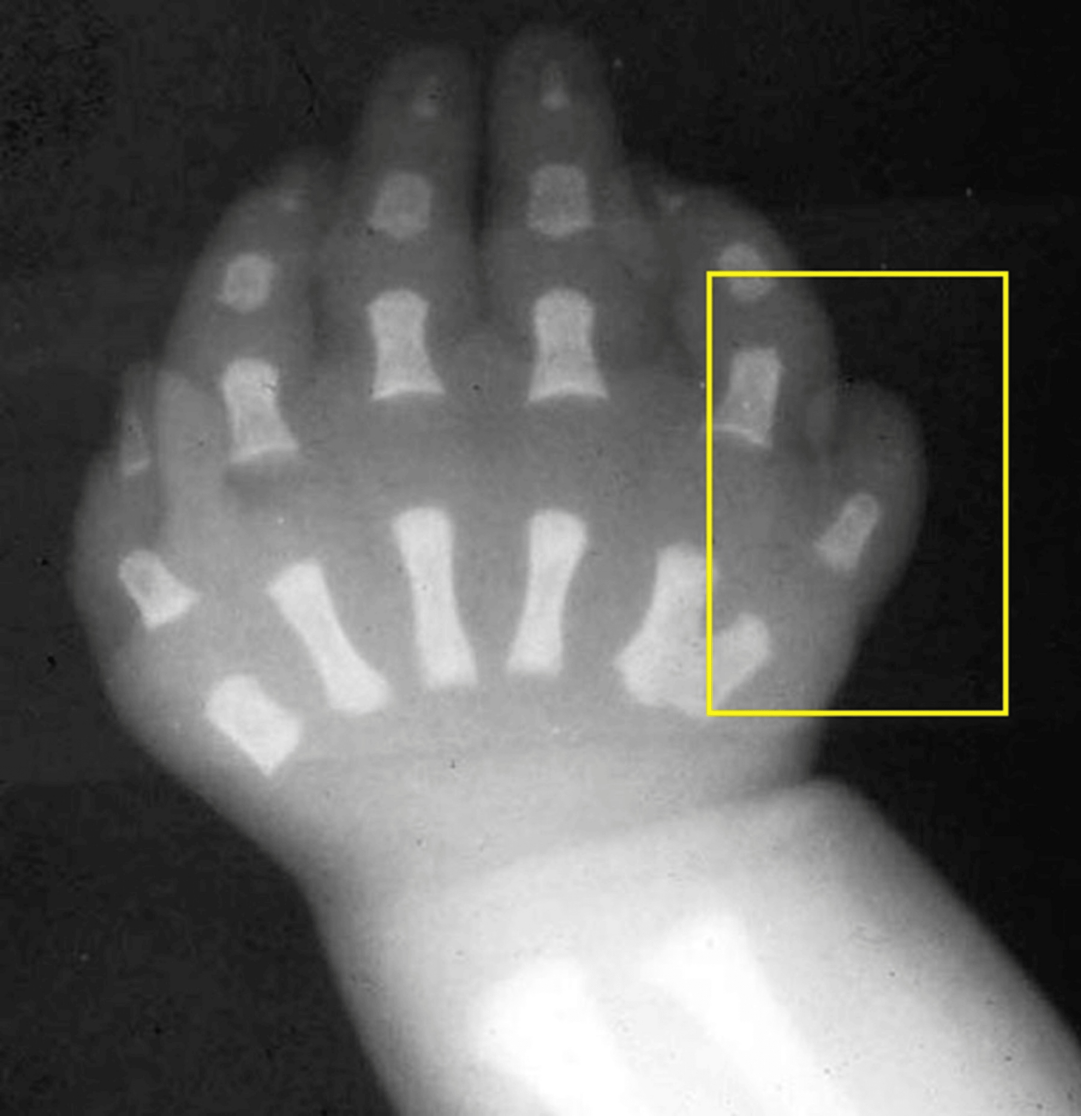
X-ray image of the patient's left hand

The patient was further subjected to radiological assessment by X-ray imaging to assess the underlying bone structure and the presence of any associated abnormalities (Figure 2).

**Figure 3:**
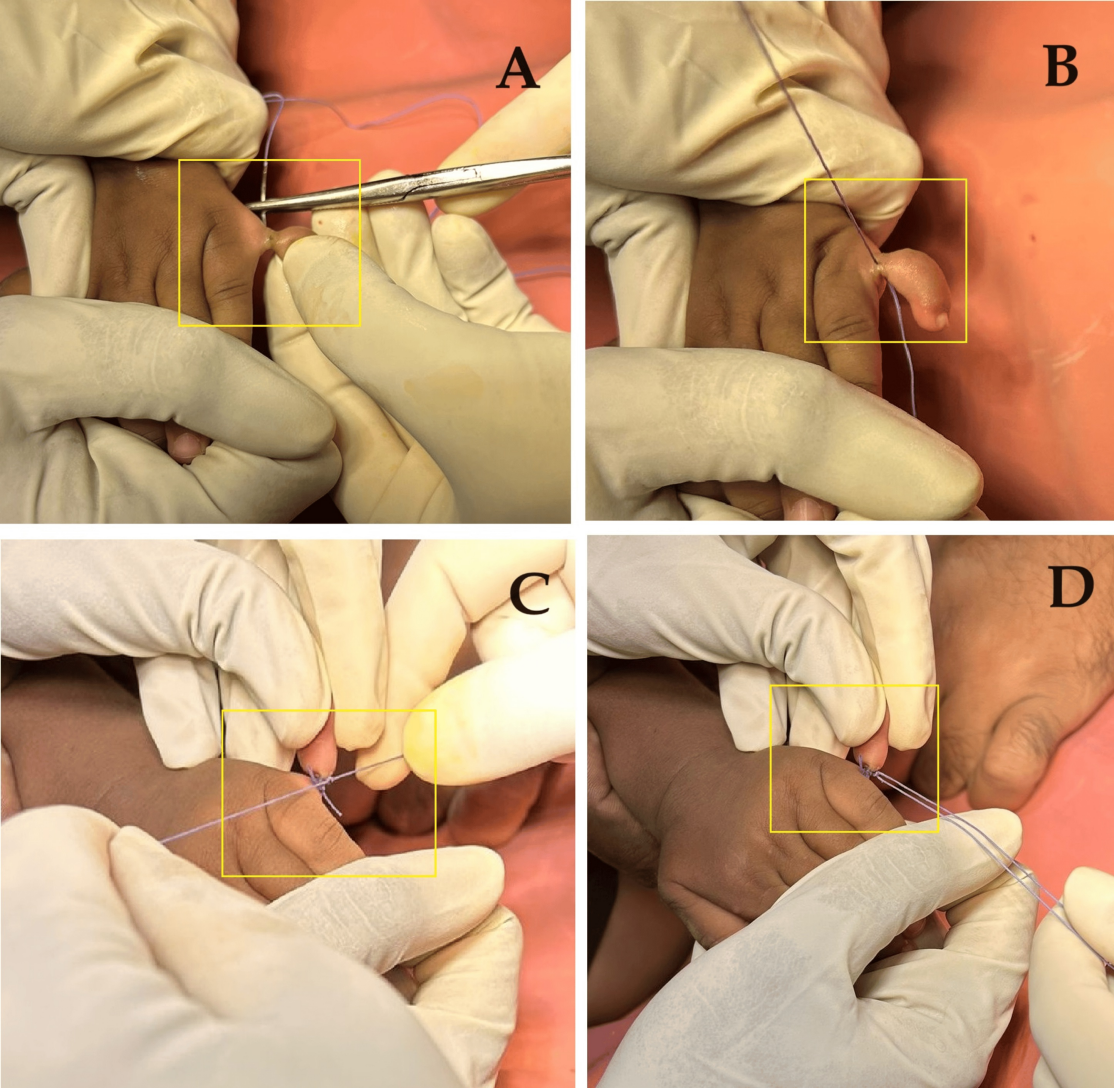
Intra-operative image of the procedure A-D: Image panes showing different stages of the removal of the extra digit.

**Figure 4:**
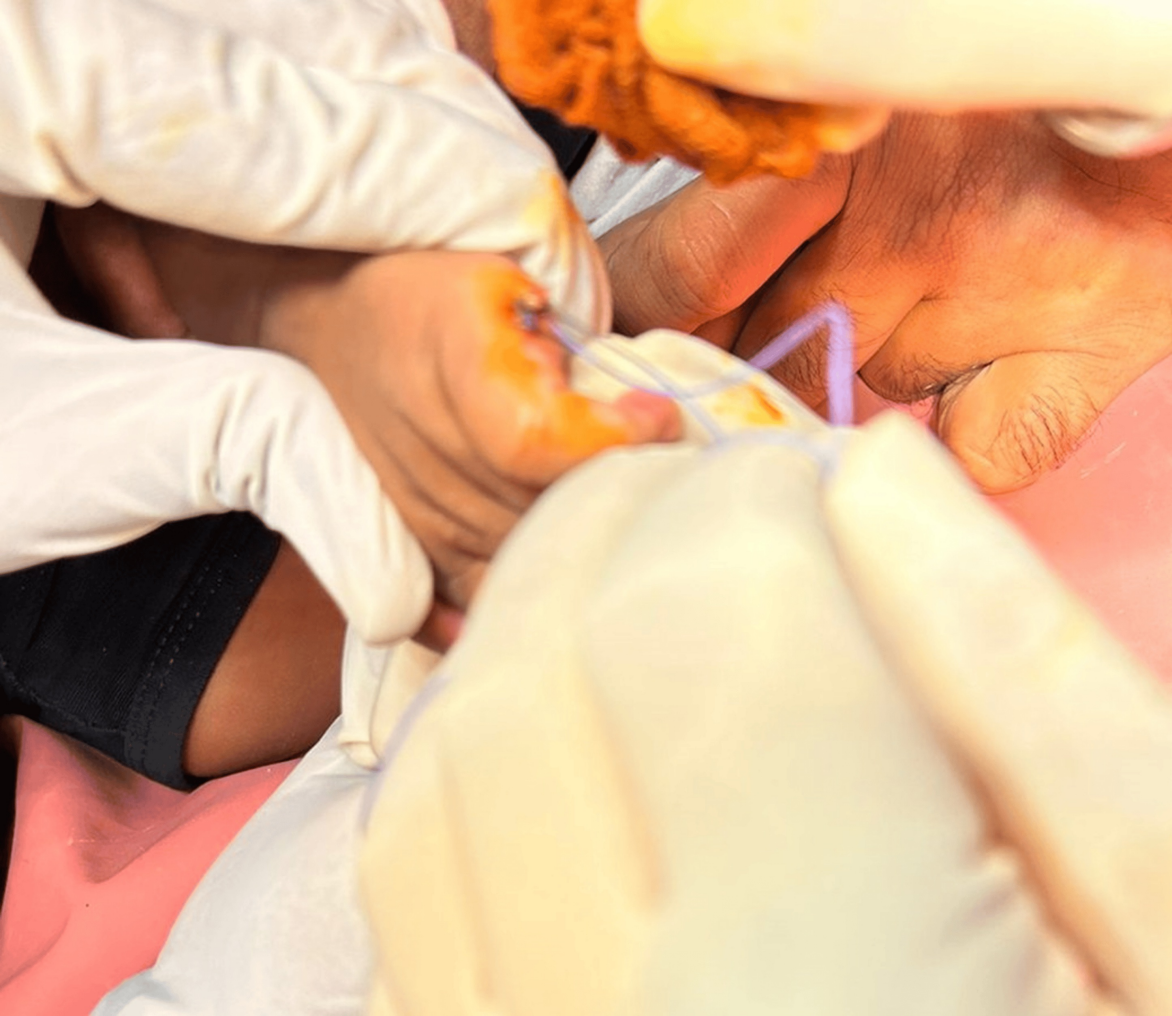
Post-operative image after the completion of the excision

**Figure 5:**
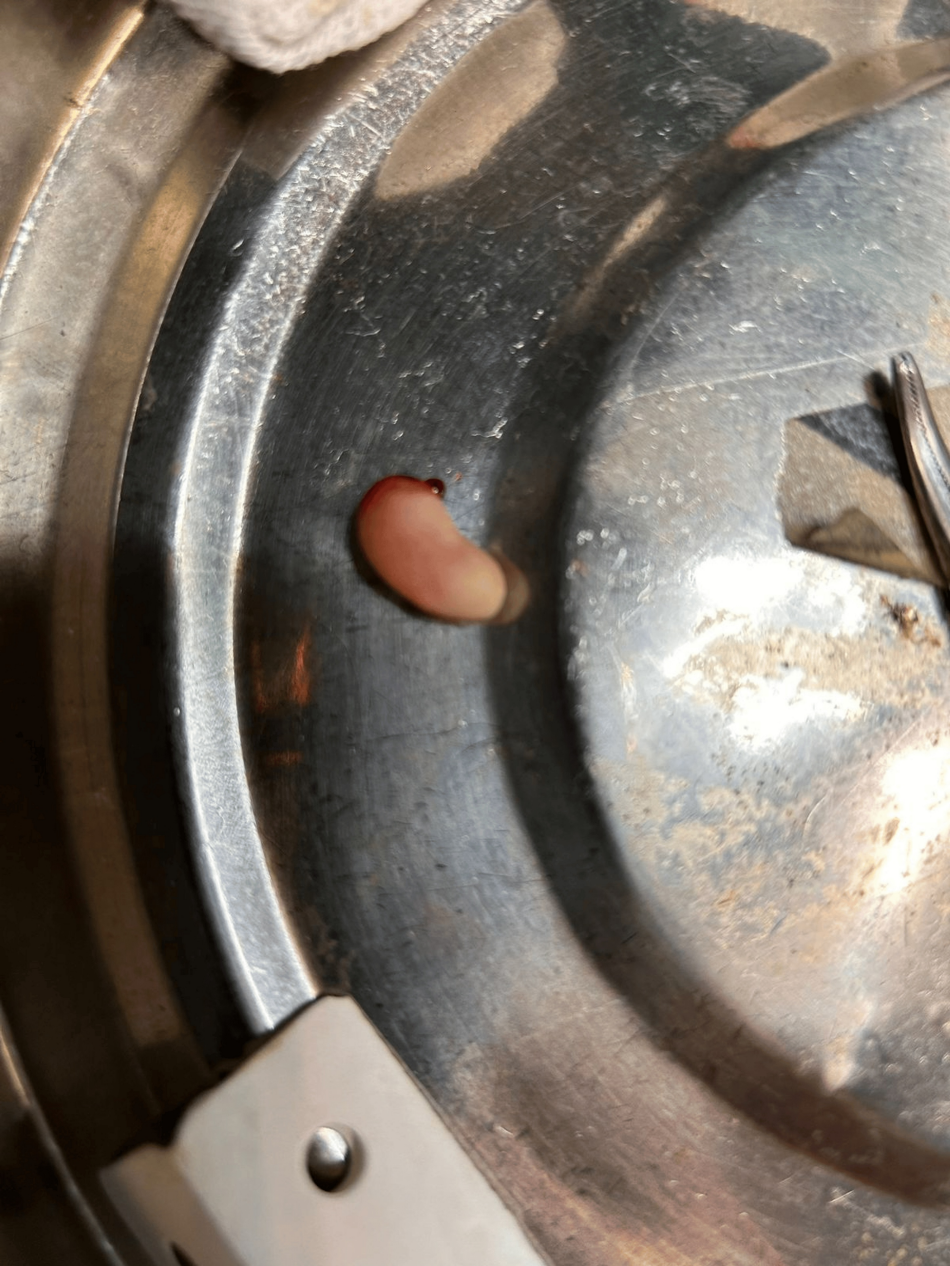
Excised specimen

The parents were counselled on the condition of the child and it was planned for surgical excision of the extra digit. The decision to perform the excision under local anesthesia was made due to the patient's age and the simplicity of the procedure. A pre-aesthetic evaluation was done, and anesthesia was administered to ensure the patient remained comfortable and immobile during the surgery. The type of anesthesia used was local anesthesia plus sedation and the procedure was conducted in an outpatient setting. A digital block using 1% lidocaine without epinephrine was administered to the base of the supernumerary digit. Once anesthesia was confirmed, a sterile technique was used to excise the extra digit using a ligature and surgical scissors. Hemostasis was achieved with minimal bleeding, and a small absorbable suture was used to close the wound. The polydactyly was corrected by the extra digit removal by amputation. The nerve and blood vessel integrity were maintained, and a cosmetically pleasing result was achieved (Figures 3-5).

Post-procedure was uneventful. The patient tolerated the procedure well and was monitored closely in the recovery area until awake and stable. Pain management and wound care protocols were implemented to ensure optimal healing. The patient was advised physical therapy and/or occupational therapy to aid in hand function and rehabilitation. The patient was scheduled for monthly follow-up appointments to monitor healing progress, assess hand function, and address any concerns or complications that may arise. Rehabilitation exercises and activities were prescribed to promote strength, flexibility, and coordination in the hand. The patient had full range of motion and normal functionality of the hand at follow-up. The parents expressed satisfaction with the cosmetic and functional outcomes.

## Discussion

Polydactyly is a genetic disorder related to the physical abnormality of the limbs. It has been characterized majorly based on osseous and non-osseous connections of the extra digits of the hands and feet [1,3]. Polydactyly can be observed in isolated forms or as a part of other syndromes. It has also been characterized as preaxial, mesoaxial, or central polydactyly and postaxial on the anatomical position of the extra digit. 

Preaxial classification can be mentioned if the extra digit is present on the radial side of the hand and the tibial (medial) side of the limbs. This is the second most common type of polydactyly based on an incidence rate of 0.8-2.3/ 10000 live births [1,5]. The presence of one extra digit is reported normally in preaxial type, but there are cases with the presence of more than 1 supernumerary digit. Though, these polydactyly forms are autosomal dominant traits, autosomal recessive traits have also been reported in the recent past found to be associated [6].

Mesoaxial or central polydactyly is defined as the digit duplication of the 2nd, 3rd, and 4th digits of the hands or the feet. This is rare amongst the three polydactyly classes. It is observed in 6% of the cases. Surgical correction in these cases is complicated in comparison to the preaxial and postaxial locations [7,8].

Postaxial polydactyly is characterized as the extra digits present after the last digit of the hands and limbs with a varying prevalence noted between 1/100 to 1/3300 based on ethnicities. This is the most common type of polydactyly noted [1,9].

Postaxial polydactyly was noted in our case, with an extra digit as an overhanging after the fifth digit of the child. Management is usually carried out by surgical removal of the extra digits in pre- and post-axial polydactyly presentations, in cases without osseous involvement. Surgical treatment procedures include vascular clipping, ligation, and surgical excision [7,10]. Removal of the postaxial supernumerary digit can be carried out under the influence of local anesthesia, by occluding the blood flow at the base of the extra digit in children [10]. This case was managed in similarity to Bjorklund and O’Brien [9], under local anesthesia by tying the base of the supernumerary digit with suture material, which was the result of blood flow obstruction and removal of the extra digit. This technique has been reported to have lesser post-operative complications such as necrosis, residual bumps, neuromas, and also, the risk associated with general anesthesia. Though the patients were less than 12 months of age [10,11]. Surgeries of older children might require general anesthesia and more complex processes. Though there are numerous genetic mutations related to postaxial polydactyly, the parents of our patient were not willing to undergo genetic screening to check for the associated cause [1,5,8]. Hence, we were not able to identify the underlying genetic cause in this case.

## Conclusions

Postaxial polydactyly is common with multiple genetic mutations and both syndromic and non-syndromic forms. Management involves surgical removal of extra digits, with treatment procedures including vascular clipping, ligation, and surgical excision. Postaxial polydactyly can be managed under local anesthesia, but in our case, genetic screening was not possible due to the parents' unwillingness to undergo genetic screening.
